# West Nile Virus and Other Arboviral Diseases — United States, 2012

**Published:** 2013-06-28

**Authors:** Nicole P. Lindsey, Jennifer A. Lehman, J. Erin Staples, Marc Fischer

**Affiliations:** Div of Vector-Borne Diseases, National Center for Emerging and Zoonotic Infectious Diseases, CDC

Arthropod-borne viruses (arboviruses) are transmitted to humans primarily through the bites of infected mosquitoes and ticks. West Nile virus (WNV) is the leading cause of domestically acquired arboviral disease in the United States (1). However, several other arboviruses also cause sporadic cases and seasonal outbreaks of neuroinvasive disease (e.g., meningitis, encephalitis, and acute flaccid paralysis) (1). In 2012, CDC received reports of 5,780 nationally notifiable arboviral disease cases (excluding dengue). A large multistate outbreak of WNV disease accounted for 5,674 (98%) of reported cases, the highest number reported since 2003. Other reported etiologies included Eastern equine encephalitis virus (EEEV), Powassan virus (POWV), St. Louis encephalitis virus (SLEV), and California serogroup viruses such as La Crosse virus (LACV) and Jamestown Canyon virus (JCV). Arboviruses continue to cause serious illness in substantial numbers of persons in the United States. Maintaining surveillance remains important to identify outbreaks and guide prevention efforts.

In the United States, most arboviruses are maintained in transmission cycles between arthropods and vertebrate hosts (typically birds or small mammals). Humans usually become infected when bitten by infected mosquitoes or ticks. Person-to-person transmission occurs rarely through blood transfusion and organ transplantation. The majority of human arboviral infections are asymptomatic. Symptomatic infections most often manifest as a systemic febrile illness and, less commonly, as neuroinvasive disease. Most endemic arboviral diseases are nationally notifiable and are reported to CDC through ArboNET (2,3). In addition to collecting data on human disease cases, ArboNET collects data on viremic blood donors, veterinary disease cases, and infections in mosquitoes, dead birds, and sentinel chickens. Using standard definitions, human cases with laboratory evidence of recent arboviral infection are classified as neuroinvasive disease or nonneuroinvasive disease (2). Because of the substantial associated morbidity, detection and reporting of neuroinvasive disease cases is assumed to be more consistent and complete than for nonneuroinvasive disease cases. Therefore, incidence rate calculations were limited to neuroinvasive disease cases.

In 2012, CDC received reports of 5,780 cases of nationally notifiable arboviral diseases, including those caused by WNV (5,674 cases), LACV (78), EEEV (15), POWV (seven), SLEV (three), JCV (two), and an unspecified California serogroup virus (one). Cases were reported from 1,020 (32%) of the 3,141 U.S. counties; no cases were reported from Alaska or Hawaii. Of the 5,780 total cases, 2,969 (51%) were reported as neuroinvasive disease, for a national incidence of 0.95 per 100,000 population.

A total of 5,674 WNV disease cases, including 2,873 (51%) neuroinvasive cases, were reported from 976 counties in 48 states, the District of Columbia, and Puerto Rico (Table 1). WNV disease cases peaked in late August, with 5,199 (92%) cases having illness onset during July–September. The median age of patients was 56 years (interquartile range [IQR]: 42–68 years); 3,193 (56%) were male. Overall, 3,491 (62%) patients were hospitalized, and 286 (5%) died. The median age of patients who died was 77 years (IQR: 68–84 years).

Of the 2,873 WNV neuroinvasive disease patients, 1,615 (56%) had encephalitis, 1,038 (36%) had meningitis, and 220 (8%) had acute flaccid paralysis. Among the 220 patients with acute flaccid paralysis, 183 (83%) also had encephalitis or meningitis. The national incidence of neuroinvasive WNV disease was 0.92 per 100,000 population (Table 2). States with the highest incidence rates included South Dakota (7.44 per 100,000), North Dakota (5.57), Mississippi (3.45), Louisiana (3.37), and Texas (3.24) (Figure). Four states reported over half of the WNV neuroinvasive disease cases: Texas (844 cases), California (297), Illinois (187), and Louisiana (155). Neuroinvasive WNV disease incidence increased with age, with the highest incidence among persons aged ≥70 years. Among patients with neuroinvasive disease, 270 (9%) died.

The 78 LACV disease cases were reported from 50 counties in 11 states; 71 (91%) were neuroinvasive (Table 1). Dates of illness onset for LACV disease cases ranged from March through October; 13 (17%) had onset during April–June, and 59 (76%) had illness onset during July–September. Forty-four (56%) patients were male. The median age of patients was 9 years (IQR: 6–13 years); 65 (83%) were aged <18 years. LACV neuroinvasive disease incidence was highest in West Virginia (0.49 per 100,000), North Carolina (0.27), Tennessee (0.14), and Ohio (0.10) (Table 2). Those four states reported 56 (79%) LACV neuroinvasive disease cases. A total of 76 (97%) patients were hospitalized; one (1%) died. Three California serogroup virus disease cases in addition to the LACV cases were reported, including two caused by JCV and one unspecified.

Fifteen EEEV neuroinvasive disease cases were reported from six states: Massachusetts (seven cases), North Carolina (two), Vermont (two), Florida (two), Georgia (one), and Virginia (one) (Table 1). Dates of illness onset ranged from June through October, with 13 (87%) occurring during July–September. The median age of patients was 57 years (IQR: 11–68 years); 13 (87%) were male. Fourteen (93%) patients were hospitalized; five (33%) died. The median age of patients who died was 76 years (IQR: 63–79 years).

Seven POWV neuroinvasive disease cases were reported from three states: Minnesota (four cases), Wisconsin (two), and New York (one) (Table 1). Dates of illness onset for all cases were in May or June. All cases occurred in adult patients (median age: 58 years [IQR: 36–73 years]); four were male. Six (86%) patients were hospitalized; none died.

Three SLEV disease cases were reported from Texas; only one was neuroinvasive. Dates of illness onset were in July and August. All cases occurred in adults aged 40–60 years. One of the three SLEV patients was hospitalized; none died.

## Editorial Note

A large multistate outbreak of WNV disease occurred in 2012, with more cases reported nationally than in any year since 2003, including the first reported human case from Maine. The 15 EEEV disease cases reported in 2012 were the most reported since 2005, and included the first cases ever reported from Vermont. EEEV disease remained the most severe domestic arboviral disease, with a 33% case-fatality rate. Over 90% of arboviral disease cases occurred during July–September, and most of the remainder occurred during April–June, emphasizing the importance of focusing public health interventions on these periods.

The national incidence of WNV neuroinvasive disease peaked in 2002 (1.02 per 100,000) and 2003 (0.98) (3). During 2004–2011, annual incidence was relatively low (median: 0.31; range: 0.13–0.50) (3–6). In 2012, the national incidence of WNV neuroinvasive disease increased to 0.92 per 100,000. The increase in disease was widespread, with 43 states reporting a higher incidence in 2012 compared with the median for 2004–2011; however, more than half of the neuroinvasive disease cases in 2012 were reported from just four states, and 29% were reported from Texas alone. Of the five states with a lower incidence in 2012 compared with the previous 8 years, four were in the Mountain Region (Montana, Nevada, Utah, and Wyoming). Oregon also reported lower rates compared with recent years; Alaska and Hawaii have never reported a case of WNV disease.

Reported numbers of arboviral disease cases vary from year to year. It is not clear why more WNV activity occurred this year than in recent years. The weather, numbers of birds that maintain the virus, numbers of mosquitoes that spread the virus, and human behavior are all factors that can influence when and where outbreaks occur. Because of this complex ecology, it is difficult to predict how many cases of disease might occur in the future and in what areas.

The findings in this report are subject to at least two limitations. First, ArboNET is a passive surveillance system that relies on clinicians to consider the diagnosis of an arboviral disease and obtain appropriate diagnostic test results and on health-care providers and laboratories to report laboratory-confirmed cases to public health authorities. Second, testing and reporting are incomplete, leading to a substantial underestimate of the actual number of cases (7). Based on previous studies, for every reported case of WNV neuroinvasive disease, there are an estimated 30–70 nonneuroinvasive disease cases. Extrapolating from the 2,873 WNV neuroinvasive disease cases reported, an estimated 86,000–200,000 nonneuroinvasive disease cases might have occurred in 2012. However, only 2,801 (1%–3%) were diagnosed and reported.

Arboviruses continue to cause severe illness in substantial numbers of persons in the United States. However, cases occur sporadically, and the epidemiology varies by virus and geographic area. Surveillance is essential to identify outbreaks and guide prevention efforts aimed at reducing the incidence of these diseases. Health-care providers should consider arboviral infections in the differential diagnosis of cases of aseptic meningitis and encephalitis, obtain appropriate specimens for laboratory testing, and promptly report cases to public health authorities (2). Because human vaccines against domestic arboviruses are not available, prevention of arboviral disease depends on community and household efforts to reduce vector populations (e.g., applying insecticides and reducing mosquito breeding sites), personal protective measures to decrease exposure to mosquitoes and ticks (e.g., use of repellents and wearing protective clothing), and screening blood donors. Updated guidelines for WNV surveillance, prevention, and control are available online from CDC at http://www.cdc.gov/westnile/resources/pdfs/wnvguidelines.pdf.

What is already known on this topic?

West Nile virus (WNV) is the leading cause of domestically acquired arboviral disease in the United States. However, several other arboviruses can cause sporadic cases and outbreaks of neuroinvasive disease, mainly in the summer.

What is added by this report?

A large multistate outbreak of WNV disease occurred in 2012. The 5,674 cases reported nationally were the highest number of cases reported since 2003. Eastern equine encephalitis, although rare, remained the most severe arboviral disease, with a 33% case-fatality rate.

What are the implications for public health practice?

WNV and other arboviruses continue to be a source of severe illness each year for substantial numbers of persons in the United States. Maintaining surveillance remains important to identify outbreaks and guide prevention efforts.

## Figures and Tables

**FIGURE:**
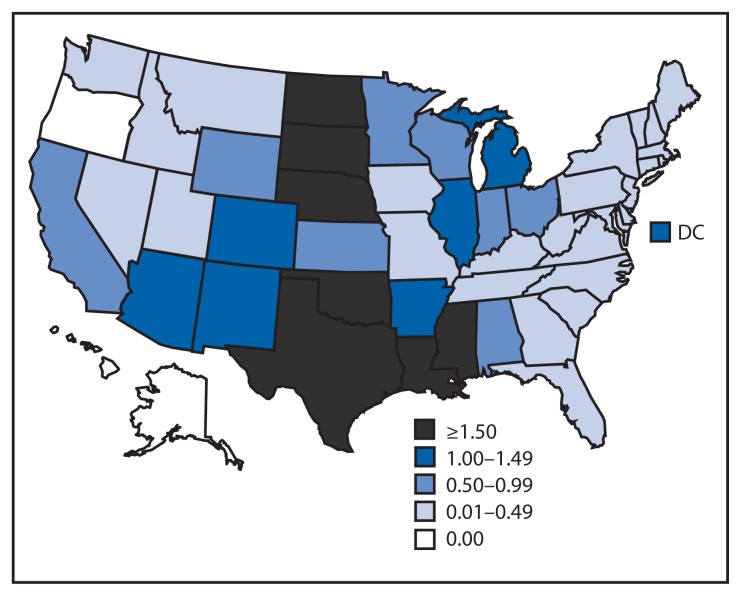
Rate^*^ of reported cases of West Nile virus neuroinvasive disease, by state — United States, 2012 ^*^ Per 100,000 population, based on July 1, 2012 U.S. Census population estimates.

**TABLE 1:** Number and percentage of reported cases of arboviral disease, by virus and selected characteristics — United States, 2012*

Characteristic	Virus							

	West Nile (N = 5,674)		La Crosse (N = 78)		Eastern equine encephalitis (N = 15)		Powassan (N = 7)	
			
	No.	(%)	No.	(%)	No.	(%)	No.	(%)
**Age group (yrs)**								
<18	210	(4)	65	(83)	4	(27)	0	—
18–59	3,124	(55)	11	(14)	4	(27)	4	(57)
≥60	2,340	(41)	2	(3)	7	(47)	3	(43)
**Sex**								
Male	3,193	(56)	44	(56)	13	(87)	4	(57)
Female	2,481	(44)	34	(44)	2	(13)	3	(43)
**Period of illness onset**								
January–March	2	(<1)	1	(1)	0	—	0	—
April–June	130	(2)	13	(17)	1	(7)	7	(100)
July–September	5,199	(92)	59	(76)	13	(87)	0	—
October–December	343	(6)	5	(6)	1	(7)	0	—
**Clinical syndrome**								
Nonneuroinvasive	2,801	(49)	7	(9)	0	—	0	—
Neuroinvasive	2,873	(51)	71	(91)	15	(100)	7	(100)
Encephalitis	1,615	(28)	56	(72)	13	(87)	4	(57)
Meningitis	1,038	(18)	14	(18)	2	(13)	3	(43)
Acute flaccid paralysis†	220	(4)	1	(1)	0	—	0	—
**Outcome**								
Hospitalization	3,491	(62)	76	(97)	14	(93)	6	(86)
Death	286	(5)	1	(1)	5	(33)	0	—

* Three California serogroup virus disease cases in addition to the La Crosse virus disease cases were reported, including two caused by Jamestown Canyon virus and one unspecified.

† Of the 220 West Nile virus disease patients with acute flaccid paralysis, 183 (83%) also had encephalitis or meningitis. The La Crosse virus disease patient with acute flaccid paralysis also had encephalitis.

**TABLE 2:** Number and rate* of reported cases of arboviral neuroinvasive disease, by virus, U.S. Census region, and state — United States, 2012

U.S. Census region and state	Virus							

	West Nile		La Crosse		Eastern equine encephalitis		Powassan	
			
	No.	Rate	No.	Rate	No.	Rate	No.	Rate
**United States**	**2,873**	**0.92**	**71**	**0.02**	**15**	**<0.01**	**7**	**<0.01**
**New England**	**42**	**0.29**	**—**	**—**	**9**	**0.06**	**—**	**—**
Connecticut	12	0.33	—	—	—	—	—	—
Maine	1	0.08	—	—	—	—	—	—
Massachusetts	25	0.38	—	—	7	0.11	—	—
New Hampshire	1	0.08	—	—	—	—	—	—
Rhode Island	2	0.19	—	—	—	—	—	—
Vermont	1	0.16	—	—	2	0.32	—	—
**Middle Atlantic**	**116**	**0.28**	**—**	**—**	**—**	**—**	**1**	**<0.01**
New Jersey	22	0.25	—	—	—	—	—	—
New York	61	0.31	—	—	—	—	1	0.01
Pennsylvania	33	0.26	—	—	—	—	—	—
**East North Central**	**494**	**1.06**	**16**	**0.03**	**—**	**—**	**2**	**<0.01**
Illinois	187	1.45	—	—	—	—	—	—
Indiana	46	0.70	2	0.03	—	—	—	—
Michigan	141	1.43	—	—	—	—	—	—
Ohio	76	0.66	12	0.10	—	—	—	—
Wisconsin	44	0.77	2	0.03	—	—	2	0.03
**West North Central**	**225**	**1.08**	**4**	**0.02**	**—**	**—**	**4**	**0.02**
Iowa	11	0.36	—	—	—	—	—	—
Kansas	20	0.69	—	—	—	—	—	—
Minnesota	34	0.63	4	0.07	—	—	4	0.07
Missouri	17	0.28	—	—	—	—	—	—
Nebraska	42	2.26	—	—	—	—	—	—
North Dakota	39	5.57	—	—	—	—	—	—
South Dakota	62	7.44	—	—	—	—	—	—
**South Atlantic**	**185**	**0.30**	**38**	**0.06**	**5**	**<0.01**	**—**	**—**
Delaware	2	0.22	—	—	—	—	—	—
District of Columbia	8	1.27	—	—	—	—	—	—
Florida	52	0.27	—	—	2	0.01	—	—
Georgia	46	0.46	—	—	1	0.01	—	—
Maryland	25	0.42	—	—	—	—	—	—
North Carolina	7	0.07	26	0.27	2	0.02	—	—
South Carolina	20	0.42	1	0.02	—	—	—	—
Virginia	20	0.24	2	0.02	1	0.01	—	—
West Virginia	5	0.27	9	0.49	—	—	—	—
**East South Central**	**173**	**0.93**	**10**	**0.05**	**—**	**—**	**—**	**—**
Alabama	38	0.79	—	—	—	—	—	—
Kentucky	13	0.30	—	—	—	—	—	—
Mississippi	103	3.45	1	0.03	—	—	—	—
Tennessee	19	0.29	9	0.14	—	—	—	—
**West South Central**	**1,146**	**3.08**	**3**	**0.01**	**—**	**—**	**—**	**—**
Arkansas	44	1.49	—	—	—	—	—	—
Louisiana	155	3.37	—	—	—	—	—	—
Oklahoma	103	2.70	—	—	—	—	—	—
Texas	844	3.24	3	0.01	—	—	—	—
**Mountain**	**190**	**0.84**	**—**	**—**	**—**	**—**	**—**	**—**
Arizona	87	1.33	—	—	—	—	—	—
Colorado	62	1.20	—	—	—	—	—	—
Idaho	5	0.31	—	—	—	—	—	—
Montana	1	0.10	—	—	—	—	—	—
Nevada	5	0.18	—	—	—	—	—	—
New Mexico	24	1.15	—	—	—	—	—	—
Utah	3	0.11	—	—	—	—	—	—
Wyoming	3	0.52	—	—	—	—	—	—
**Pacific**	**301**	**0.59**	**—**	**—**	**—**	**—**	**—**	**—**
Alaska	—	—	—	—	—	—	—	—
California	297	0.78	—	—	—	—	—	—
Hawaii	—	—	—	—	—	—	—	—
Oregon	—	—	—	—	—	—	—	—
Washington	4	0.06	—	—	—	—	—	—
**Territories**								
Puerto Rico	1	0.03	—	—	—	—	—	—

* Per 100,000 population, based on July 1, 2012 U.S. Census population estimates.
